# Potential of *Zymomonas mobilis* as an electricity producer in ethanol production

**DOI:** 10.1186/s13068-020-01672-5

**Published:** 2020-03-05

**Authors:** Bo-Yu Geng, Lian-Ying Cao, Feng Li, Hao Song, Chen-Guang Liu, Xin-Qing Zhao, Feng-Wu Bai

**Affiliations:** 1 State Key Laboratory of Microbial Metabolism, Joint International Research Laboratory of Metabolic & Developmental Sciences of Ministry of Education, School of Life Sciences and Biotechnology, Shanghai Jiao Tong University, Shanghai, 200240 China; 2 Key Laboratory of Systems Bioengineering (Ministry of Education), School of Chemical Engineering and Technology, Tianjin University, Tianjin, 300072 China

**Keywords:** *Zymomonas mobilis*, Microbial fuel cell (MFC), Biofilm, Extracellular electron transfer, Redox balance

## Abstract

**Background:**

Microbial fuel cell (MFC) convokes microorganism to convert biomass into electricity. However, most well-known electrogenic strains cannot directly use glucose to produce valuable products. *Zymomonas mobilis*, a promising bacterium for ethanol production, owns special Entner–Doudoroff pathway with less ATP and biomass produced and the low-energy coupling respiration, making *Z. mobilis* a potential exoelectrogen.

**Results:**

A glucose-consuming MFC is constructed by inoculating *Z. mobilis*. The electricity with power density 2.0 mW/m^2^ is derived from the difference of oxidation–reduction potential (ORP) between anode and cathode chambers. Besides, two-type electricity generation is observed as glucose-independent process and glucose-dependent process. For the sake of enhancing MFC efficiency, extracellular and intracellular strategies are implemented. Biofilm removal and addition of *c*-type cytochrome benefit electricity performance and Tween 80 accelerates the electricity generation. Perturbation of cellular redox balance compromises the electricity output, indicating that redox homeostasis is the principal requirement to reach ideal voltage.

**Conclusion:**

This study identifies potential feature of electricity activity for *Z. mobilis* and provides multiple strategies to enhance the electricity output. Therefore, additional electricity generation will benefit the techno-economic viability of the commercial bulk production for biochemicals or biofuels in an efficient and environmentally sustainable manner.

## Background

Microbial electrochemical technologies (MET) have been widely developed and applied in many fields, including microbial fuel cell (MFC), microbial electrosynthesis, and autonomous power sources for sensors, beacons, and seawater desalination. In particular, MFC is an environmentally sustainable technology that takes advantage of microorganisms as biocatalyst to produce electricity from various organic substances [1, 2].

Electro-active microorganisms are also known as exoelectrogens such as well-studied model strains *Geobacter sulfurreducens* and *Shewanella oneidensis* [3, 4]. Through electron transport chain, these bacteria pump out electrons to the environment for obtaining energy. In fact, instead of oxygen, metal ion or electrode are more likely to be final electron acceptors via contact-based or shuttles-mediated extracellular electron transfer (EET) pathway. Highly effective EET enables *G. sulfurreducens* and *S. oneidensis* to be promising strains for MFC [5, 6]. Unfortunately, both of them cannot directly use the most common carbon source, glucose which resulted in limited substrate spectrum. Hence, several strategies have been demonstrated to make broad range of carbon source available for them, such as introducing exogenous pathways and co-cultivation with other strains [7–9]. Besides, neither wild type nor engineered strains has an ability to produce valuable metabolites, which restricts the application of electro-active microorganism in view of economic benefits.

*Zymomonas mobilis* is an efficient ethanol-producing strain because of its unique Entner–Doudoroff pathway with less ATP and biomass produced for more sugar to be used for ethanol production, which achieves higher observed yield and higher ethanol productivity than *Saccharomyces cerevisiae* [10, 11]. Interestingly, the special respiratory chain on membrane makes *Z. mobilis* a potential exoelectrogen. The membrane contains an active branched respiratory chain, with type II NADH dehydrogenase, coenzyme Q10, cytochrome BD and several *c*-type cytochrome as terminal oxidases, together with some minor or still unidentified constituents [12, 13]. Although ethanol fermentation and respiration are the major consumers of NADH in *Z. mobilis* catabolism, this sort of respiration chain with low-energy coupling serves for more functions than oxidative phosphorylation and ATP production. Besides, unlike yeast, transition from anaerobic to aerobic growth conditions cannot improve *Z. mobilis* biomass yield [14, 15], revealing that oxygen is not the indispensable electron acceptor. Hence, low-energy coupling respiratory chain may generate electricity by offering electrode as an electron acceptor in MFC.

Oxidation-reduction potential (ORP) is an indicator of electron activities during fermentation, which provides a real-time information about redox status of external environment [16, 17]. Commonly, high ORP indicates an oxidative status and low ORP signifies a reductive status. A significant ORP decline was observed during *Z. mobilis* fermentation due to reducing external environment formed via cell metabolism. Thus, the ORP difference between cathode chamber within oxidative solution and anode chamber within *Z. mobilis* becomes a kind of driving force to generate the electrical energy. Simultaneously, *Z. mobilis* is able to convert glucose to electrical energy partially without complicated genetic engineering technologies. Although an electrochemical analysis has been done in a cell-free system of *Z. mobilis*, electricity generation ability of whole cell has not been measured so far [18].

In this study, the electrochemical performance of *Z. mobilis* was evaluated during ethanol production, and some strategies were also undertaken to improve the voltage output, such as removal of biofilm, EET pathway enhancement and perturbation of intracellular redox balance.

## Results and discussion

### Electricity generation by *Z. mobilis* ZM4

**Fig. 1:**
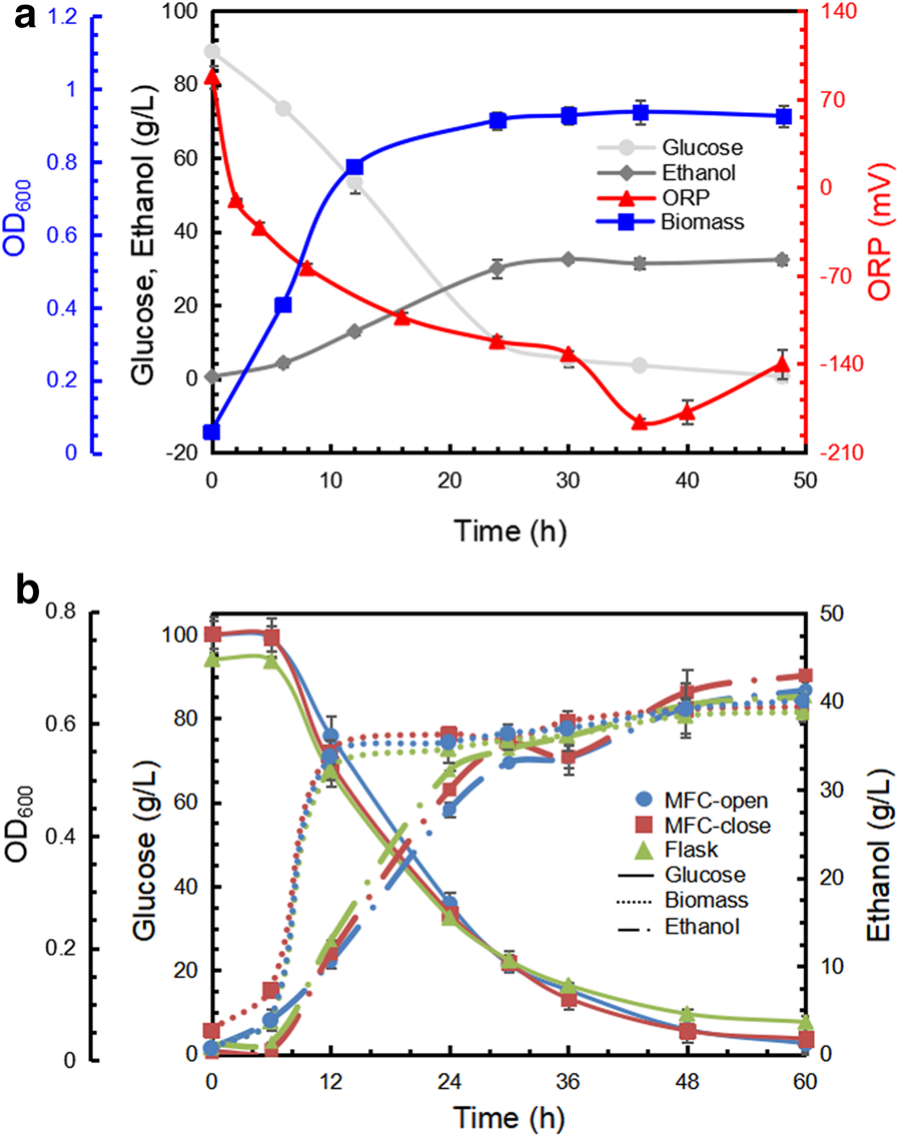
Profile of ORP, glucose, ethanol and biomass in ethanol fermentation by *Z. mobilis* (**a**) and the comparison of cell growth and fermentation in MFCs and flasks (**b**)

During ethanol fermentation by *Z. mobilis* ZM4, the ORP value of broth kept decreasing in the initial 36 h, followed by a slight recovery until 48 h (Fig. 1a). It has been reported that the glucose consumption rate is the main cause for the altered ORP [19]. Before 36 h, rapid glucose consumption, attributed to active metabolism, tended to release and accumulate the reducing power from the substrate, which consequently pull down the ORP value. Afterward, cell lysis started the oxidized compounds release, which led to a little bit restoration of ORP. Therefore, *Z. mobilis* has potential to build up a reducing environment and form the ORP difference to produce electricity in MFC. Besides, Ethanol production was monitored in an open circuit MFC, a closed circuit MFC, and flasks. Because of no significant difference among these conditions, electricity generation showed no competition with ethanol production for ZM4 (Fig. 1b). Moreover, it can be speculated that although oxygen may affect the quantity of ethanol, the independent relationship between ethanol production and electricity generation makes *Z. mobilis*-inoculated MFC less sensitive to oxygen compared to yeast-based biofuel cells [20].

**Fig. 2:**
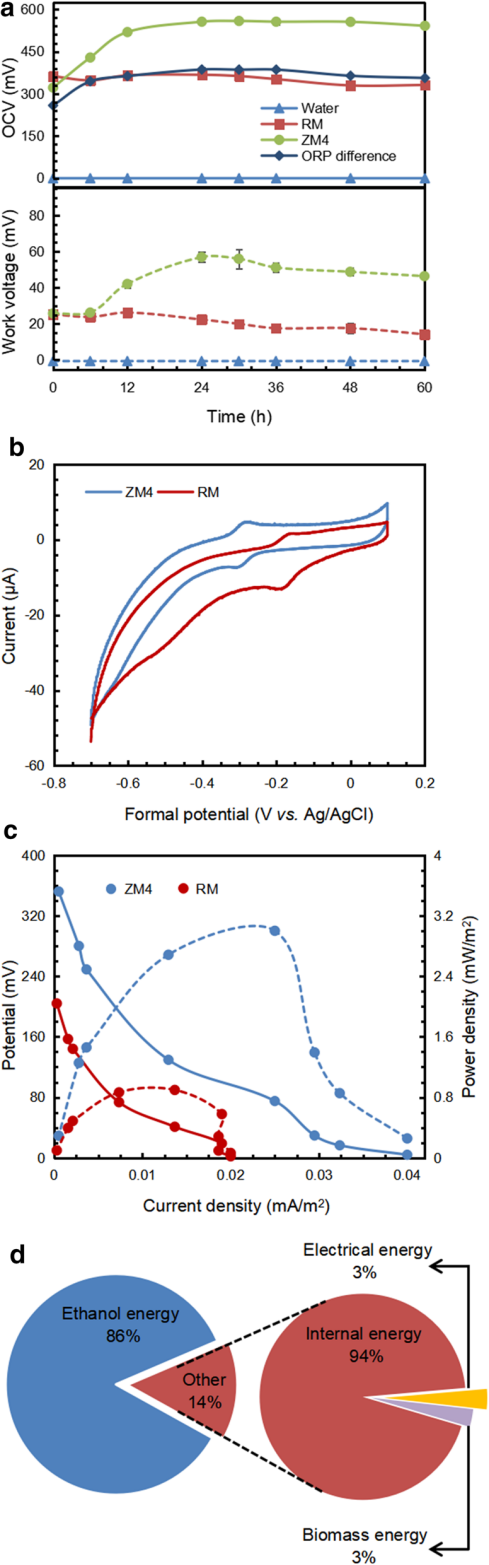
ORP difference (solid line) between anode and cathode chambers during bioethanol production by *Z. mobilis* and electricity performance comparison among water, RM medium, and *Z. mobilis* ZM4 (OCV: solid lines, WV: dotted lines) (**a**), cyclic voltammetry measurement (**b**), polarization (solid lines) and power curves (dotted lines) of *Z. mobilis*-inoculated MFC (**c**) and energy flow during power generation of *Z. mobilis* (**d**)

To further identify the potential capacity of electricity generation for *Z. mobilis* ZM4, OCV and WV are shown in Fig. 2a. Compared to medium-loaded MFC with stable voltage, ZM4-inoculated MFC exhibited a significant voltage output, which increased rapidly and peaked at 30 h, and then kept at high level. The maximal WV was about three-fold higher than that of the medium-loaded MFC, which meant that ZM4 was able to yield more electrons to enhance electricity generation. Moreover, a previous research showed that the electrical outputs are connected with the bacterial culture development [21]. And there is a close connection between ORP and bacterial growth. ORP curve resembled the OCV and WV curves indicating that compared to bacterial growth, ORP is a more direct factor to promote electricity generation.

However, due to inefficient electron generation and transfer, the voltage output of ZM4 was relative lower than other well-known exoelectrogens [3, 4].

Cyclic voltammetry was conducted to reveal the redox reaction that occurred in the equipment. As shown in Fig. 2b, a prominent redox peak appeared around − 0.18 V in medium-loaded MFC while the redox peak moved to around − 0.29 V in ZM4-inoculated MFC, which was caused by the direct involvement of conductive *c*-type cytochromes [22]. These changed peaks indicated that the mechanism of extracellular electron transfer was different from medium-mediated ones. To further investigate electrochemical performance, the polarization and power curves were obtained (Fig. 2c). Compared with the power generated in uninoculated MFC, ZM4-inoculated MFC exhibited a two-fold higher maximum power density of approximately 2.0 mW/m^2^.

An energy balance was calculated to clarify the energy flow during power generation and ethanol production. Approximately 86% of the total energy was captured by ethanol (Fig. 2d). By establishing the MFC equipment, approximately 3% of the energy was derived from the internal energy in the form of electricity. Compared with *Shewanella oneidensis*, ZM4’s recovered electrical energy was 80-fold lower [22]. But considering the energy captured by ethanol, the total generated energy of ZM4 was still significant.

### The mechanism of electricity generation

**Fig. 3:**
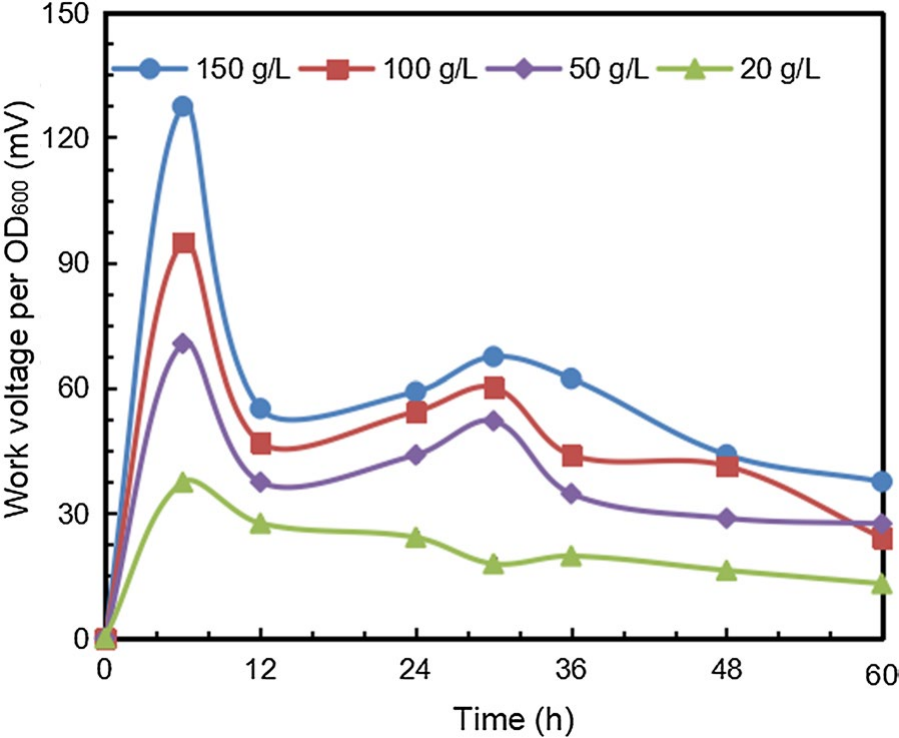
Specific work voltage at different glucose concentrations

According to Fig. 3, the specific WV (work voltage per OD_600_) varied with the concentration of glucose. As an electron donor of ZM4-inoculated MFC, a higher glucose concentration would yield more electrons. Hence, MFC with 150 g/L glucose exhibited the highest spcific WV 72.3 mV, while as glucose decreased to 20 g/L, the specific WV was only 23.6 mV.

Additionally, the specific WV also varied over time. From 12 to 60 h, the specific WV increased and peaked at 30 h, followed by a slight regression. Based on the ORP monitoring results, the ORP difference was formed by reducing compounds accumulation in the broth via consuming the substrate by *Z. mobilis*. Therefore, the glucose consumption rate can be considered as a main pointer for electricity generation. Interestingly, another voltage peak appeared between 0 and 12 h, when glucose was not used at all by microorganism, indicating that electricity generation was independent of glucose utilization before 12 h. Accordingly, the entire electricity generation could be divided into two types: a glucose-independent process and a glucose-dependent process.

**Fig. 4:**
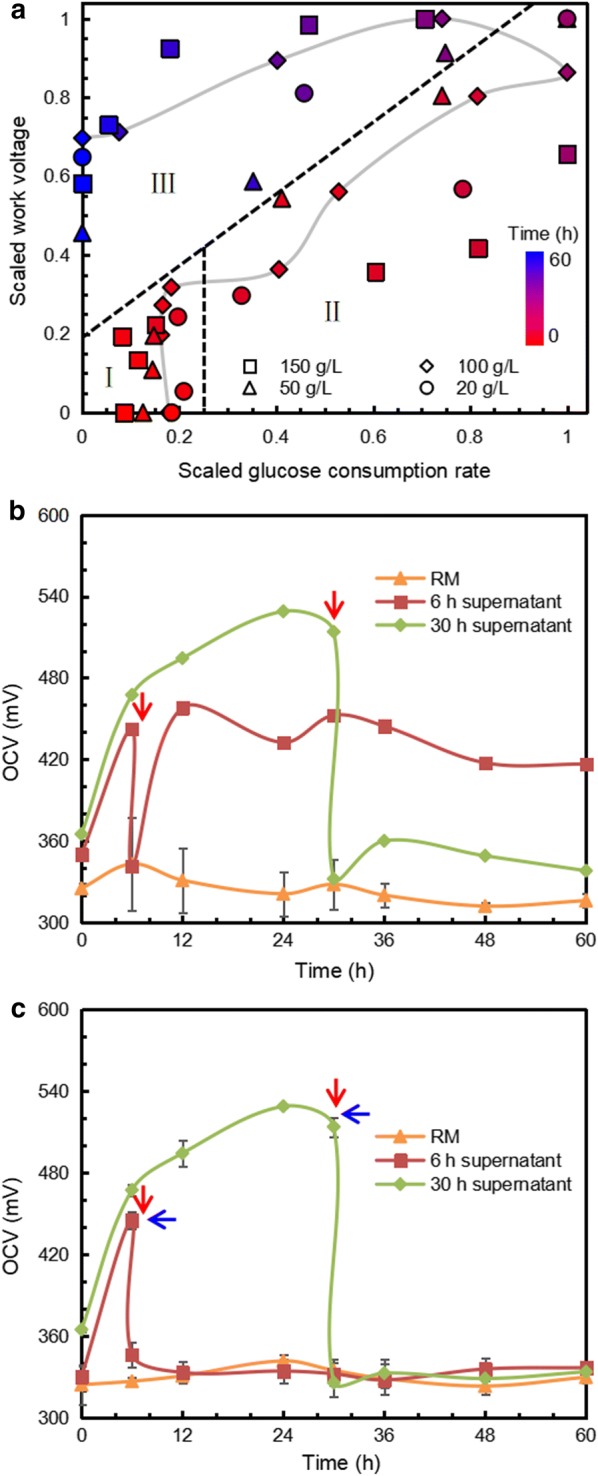
The correlation between glucose consumption rate and WV. Colors represent fermentation time and initial glucose were set as 20, 50, 100, 150 g/L, among which, 100 g/L plots were linked (**a**). The electricity generation process of RM, 6 h supernatant and 30 h supernatant (**b**, **c**). Centrifugation was implemented at the position of red arrow while the supernatants at the 6 h and 30 h of cultivation were collected from MFCs and then were loaded in new MFCs to examine their performance. Oxygen was sparged at the position of blue arrow

For better understanding of two-type electricity generation, data scaling was adopted to normalize the variable range of WV at different glucose concentrations. In the left-bottom area of Fig. 4a, the WV increased sharply when the glucose consumption rate remained unchanged, signifying that a glucose-independent electricity generation process occurred in the lag growth phase. In the right part of Fig. 4a, representing the exponential growth phase, the WV obeyed positive linearity with the glucose consumption rate. In the left-top area of Fig. 4a, ZM4 entered a stationary growth phase, when two types of electricity generation simultaneously emerged. Furthermore, scatter diagrams of other initial glucose concentrations also showed similar trends as 100 g/L, confirming a two-type process was a common phenomenon for ZM4-inoculated MFC, and that the glucose consumption rate was the direct driver of glucose-dependent electricity generation progress.

Since the increased voltage during 0–6 h was independent of glucose uptake, the supernatant should contain some chemicals that contribute to the electricity generation. To verify this hypothesis, supernatants of broth at 6 h and 30 h were added in MFC (Fig. 4b). The electricity performance of the supernatant at 6 h restored immediately, but the supernatant at 30 h maintained basal level. Hence, the glucose consumption rate was the only contributor to electricity generation at 30 h, while the supernatant was the key driving force for MFC at 6 h, as the removal of cells did not strongly influence electricity performance. Although *Z. mobilis* cells cease growth and glucose usage during 0–6 h, they continue to release reducing substances, leading to the ORP difference and voltage output. Meanwhile, those reducing substances could be depleted because of low voltage output in the supernatant without cells at 30 h.

In addition, according to Fig. 4c, once oxygen was sparged into the supernatant at 6 h, extra voltage generated by reducing substances vanished, which confirmed the fact that reducing substances contributing to electricity generation could be eliminated by oxygen. In contrast, sparging oxygen at 30 h had no influence on the electrical properties, indicating the depletion of these reducing substances. Overall, the cooperation between the reducing substances in supernatant and glucose consumption by cells contributes to the total electricity generation.

### Improvement of MFC by biofilm removal

Previous researches showed that biofilm attached on the electrode has significant influence on electricity production [23, 24]. Concerning ability of ZM4 to form biofilm on hydrophobic surfaces [25], biofilm formation on carbon cloth and evaluation of its role in electrogenesis were undertaken.

**Fig. 5:**
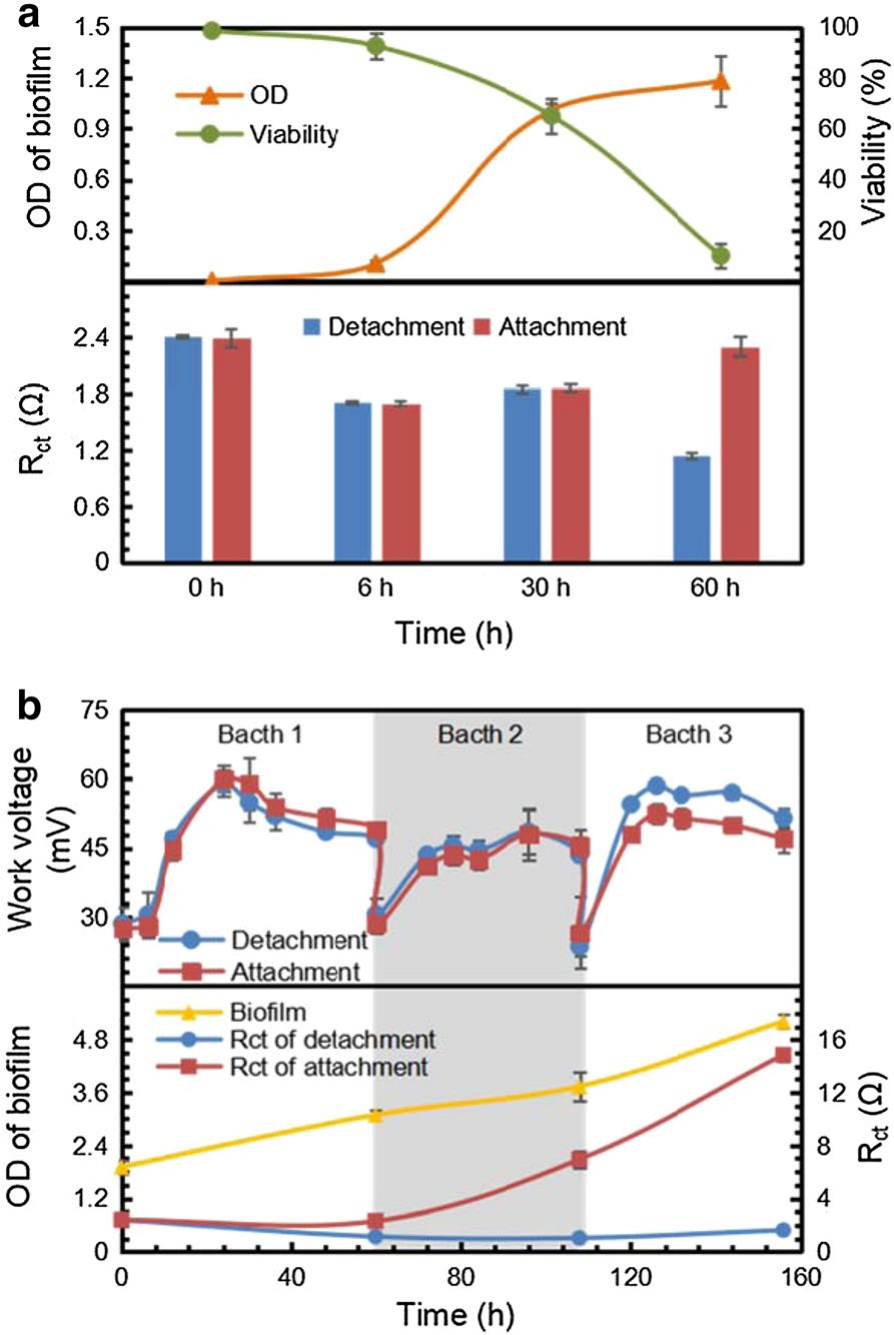
Biofilm formation on electrode surface, and charge transfer resistance on detached and attached electrode (**a**), and related electricity performance in fed-batch electricity generation process (**b**)

According to Fig. 5a, the biofilm proliferated between 6 and 30 h and remained stable after it reached the maximum. Meanwhile, the charge transfer resistance (R_ct_) of the electrode calculated by EIS decreased from 0 to 30 h. As soon as the biofilm ceased growing, R_ct_ restored to its original level. To elucidate the exact relationship between R_ct_ and the biofilm, the bacterial viability, defined as the ratio of live cells in biofilm, was quantified at different time points. Live cells dominated in the biofilm before 30 h, but as the biofilm continued growing, the number of dead cells began to increase and exceeded live cells at 60 h. R_ct_ rose as the viability of cells dropped. The dead cells were not only capable of electricity generation, but also hampered the electron transfer process on the electrode surface [26, 27]. Therefore, the rise of charge transfer resistance at the later phase results from the continuously decreasing viability.

To prevent increased resistance, the biofilm attached to the electrode was cleared at 0, 6, 30, and 60 h (Fig. 5a). There was no significant difference in R_ct_ caused by the biofilm between the attached and detached electrode until 60 h. The R_ct_ of the detached electrode was almost half of the attached electrode at 60 h, indicating that the removal of the biofilm dominated by dead cells helps enhance electron transfer to the electrode.

Due to positive effect on decreased resistance, biofilm removal was performed in three repeated batches to investigate long-term influence of biofilm on electrical performance. In each batch, the electrode was rinsed to detach biofilm when glucose was exhausted. According to Fig. 5b, in the first two batches, the electricity generation of the detachment and attachment groups were maintained at the same level. But the WV of the detachment group was 10% higher than that in the attachment group in the third batch.

Given the biofilm and charge transfer resistance, although biofilm in the attachment group grew continuously due to the replenishment of fresh medium during the first two batches, the limited quantity of biofilm remained incapable of significantly influencing resistance and electricity performance. In the third batch, the entire surface of electrode had been covered by accumulated biofilm, which interrupted the electron transfer process resulting in a sharp rise in resistance. Hence, biofilm removal increased the WV in the third batch. Moreover, extracellular polymeric substances (EPS), as the main component of biofilm, may also affect the electron transfer in biofilm. EPS have been reported to facilitate electricity generation because they contain several active groups, which may work as mediators for electron transfer from cells to electrodes [28, 29]. However, EPS consist of cellulose mainly in ZM4 [30], which may not be available for electron transfer. Besides, electrochemical characterization of biofilm in ZM4 has not been reported neither. As the removal of biofilm contributes to the decline of R_ct_, it is supposed that EPS are not electro-active and hinder electron transfer on the surface of electrode for ZM4.

However, the effect of biofilm removal is subtle due to the insufficient electricity generation ability of ZM4. Therefore, several strategies were implemented to strengthen the EET.

### Improvement of MFC by EET enhancement

**Fig. 6:**
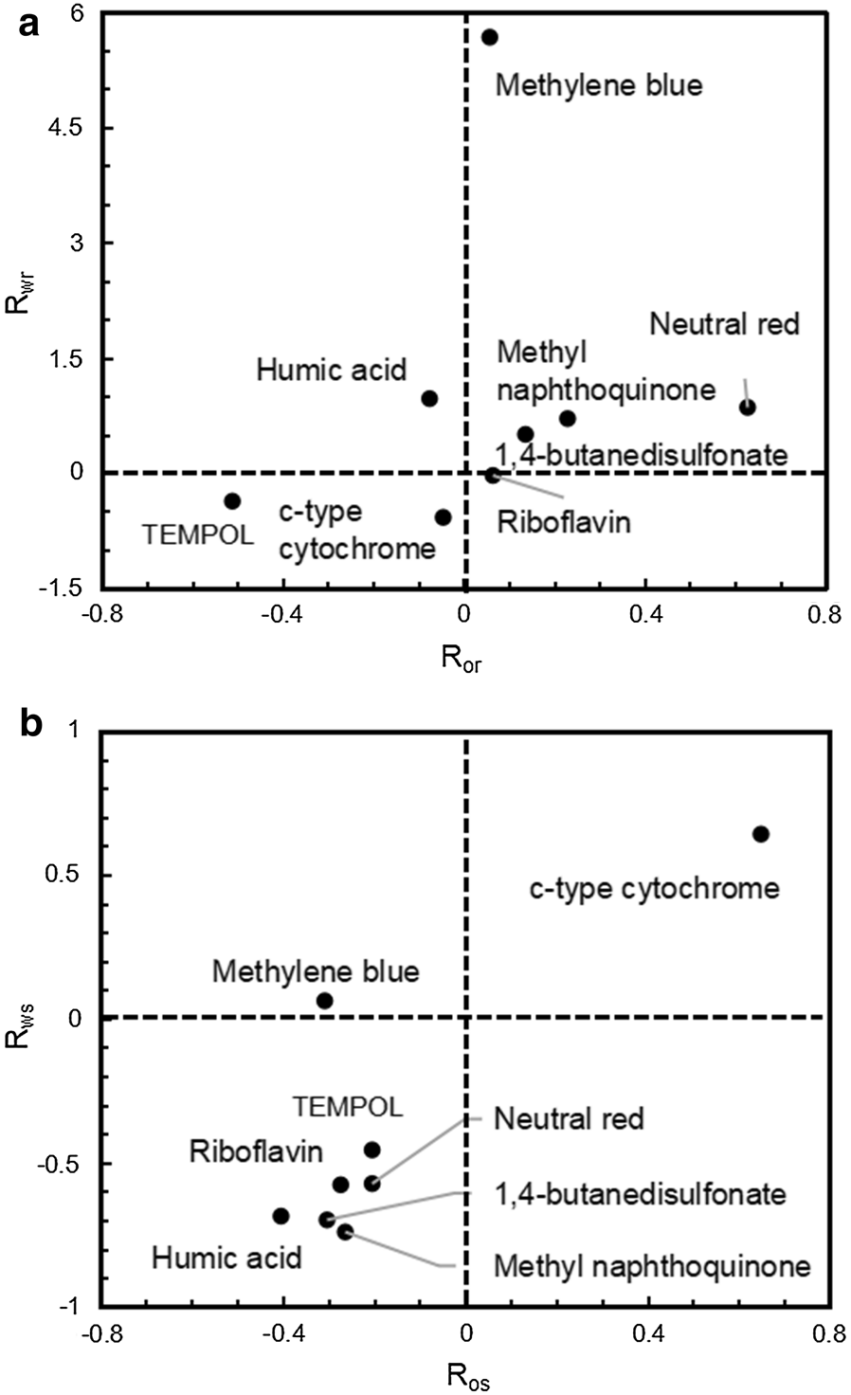
Voltage improvement ratio of reagents (**a**) and cells (**b**) (*R*_wr_: WV improvement ratio of reagents, *R*_or_: OCV improvement ratio of reagents, *R*_ws_: WV improvement ratio of cells with reagents, *R*_os_: OCV improvement ratio of cells with reagents)

Frequently, the EET pathway is a bottleneck for further improving the voltage output of exoelectrogens [31]. It consists of an indirect electron shuttle-meditated pathway [32] and the direct pathways, including appendages [33], nanowires [34] and *c*-type cytochrome [35]. Hence, adding electron shuttles and *c*-type cytochrome was implemented to enhance the EET pathway. The previous research shows that the addition of different quantity of different exogenous mediators leads to different electrical outputs [36]. The same phenomenon was also observed in this study (Additional file 1: Table S1). To exclude the influence of the medium, voltage improvement ratios of ZM4 (*R*_os_ and *R*_ws_) and reagents (*R*_or_ and *R*_wr_) were adopted to evaluate the contribution of the bacterium and the reagents to voltage, respectively. The *R*_or_, *R*_wr_ in Fig. 6a indicate the electricity performance of these mediators themselves. The dot with high values of *R*_or_ or *R*_wr_ means that the reagent itself could enhance the OCV or work voltage output, even without *Z. mobilis*. The *R*_os_, *R*_ws_ in Fig. 6b indicate the electricity performance of *Z. mobilis* with cooperation of these mediators. The dot with high values of *R*_os_ or *R*_ws_ means that the reagent helps *Z. mobilis* obtain improved electricity performance and output more OCV or work voltage.

Figure 6a shows that methylene blue and neutral red significantly enhanced the electricity performance: there was a noticeable increase on *R*_wr_ of methylene blue and *R*_or_ of neutral red. Methylene blue and neutral red helped improve work voltage and OCV outputs, respectively. However, without inoculation of ZM4, these changes could still be achieved by adding these electron shuttles only. In terms of the voltage improvement ratio of ZM4 (*R*_os_ and *R*_ws_) (Fig. 6b), the cells did not indicate an enhanced ability to generate electricity with the addition of electron shuttles that showed significant changes in Fig. 6a. Hence, for methylene blue and neutral red, the improved electricity performance resulted from the interaction between these reagents and medium. These two reagents have no effect on helping ZM4 obtain enhanced electricity generation ability. However, *c*-type cytochrome promoted ZM4 to output much more voltage (*R*_os_ and *R*_ws_ were both improved). Meanwhile, *c*-type cytochrome had less influence on MFC (*R*_or_ and *R*_wr_ remained nearly unchanged). Therefore, instead of an indirect electron shuttle-mediated pathway, enhancing direct electron transfer could significantly benefit the electricity performance, which supported the CV measurement results that the main EET mechanism is direct involvement of conductive *c*-type cytochrome.

**Fig. 7:**
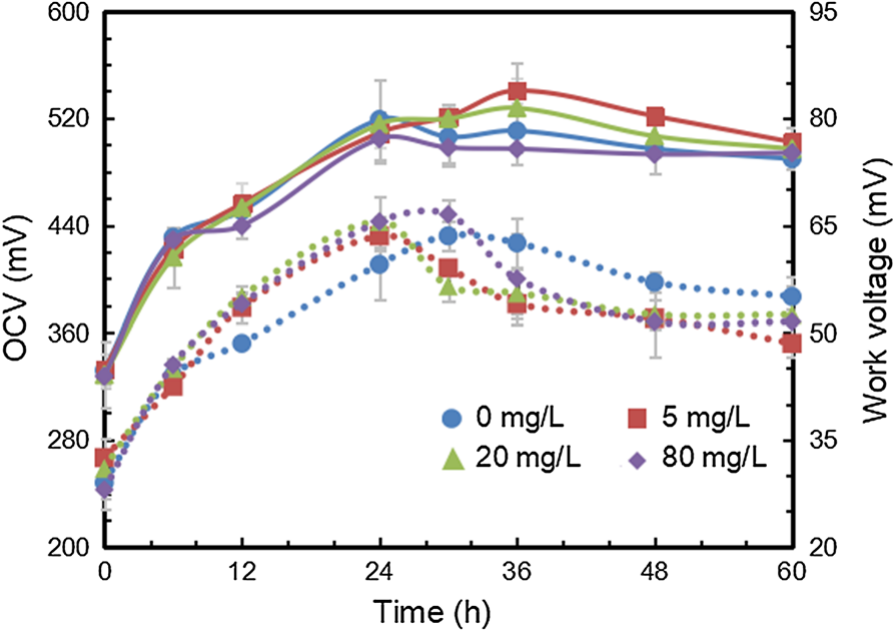
Electrical performance (OCV: solid lines, WV: dotted lines) of MFC at different concentrations of Tween 80. The left vertical axis and right vertical axis indicate the values of solid lines and dotted lines at different time points, respectively

In addition, improving cell membrane permeability was also attempted to enhance the transport of electron shuttles across cell membranes and achieve more efficient EET [37]. Tween 80 as a surfactant with concentration of 0, 5, 20, and 80 mg/L were chosen to increase permeability of ZM4 [38]. Figure 7 shows that low addition (5 and 20 mg/L) of Tween promoted the OCV after 24 h, but the highest concentration (80 mg/L) inhibited OCV. The results reflected that appropriate Tween 80 enhanced the transport of electron shuttles. On the other hand, with the addition of Tween 80, WV reached peaks earlier, but the entire power density maintained same with MFC without Tween 80 addition. Because the total electrical energy is determined by the available electron pool, which is not closely related to the surfactant. Hence, improving the total quantity of electrons is the key factor for enhancing the whole electricity generation.

### Improvement of MFC by intracellular redox homeostasis

**Fig. 8:**
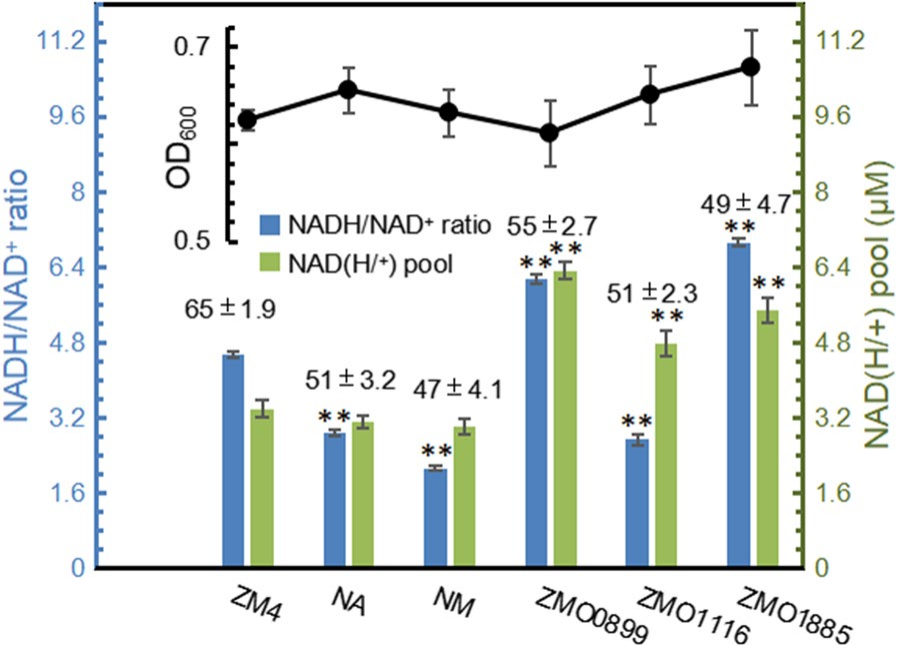
NAD(H/+) pool, NADH/NAD^+^ ratio, biomass, and maximum WV with gene overexpression and addition of NAD^+^ precursor. The data above the bar represents the maximum WV (mV) and the black line on the top of the figure compares the biomass. *p* values were calculated between ZM4 and other treatment groups to compare the difference of NAD(H/+) pool and NADH/NAD^+^ ratio (*p value < 0.05; **p value < 0.01)

Commonly, electrons were stored in cells in form of NADH [39]. In theory, increasing ratio of NADH to NAD^+^ could help elevate electron pool to enhance voltage output [40]. Adding NAD^+^ precursor nicotinic acid (NA) and nicotinamide (NM), or overexpressing cofactor related genes (*ZMO0899, ZMO1116, ZMO1885*) were adopted. Figure 8 reveals that these two methods are successful to alter ratio of NADH/NAD^+^: precursors addition and overexpressing *ZMO1116* (Glutamate synthase: reduce NAD^+^ to NADH and increase the ratio of NADH/NAD^+^) decreased the ratio while overexpressing *ZMO0899* (NAD^+^ synthase: increase the pool of NAD(H/+) and *ZMO1885* (NADH oxidase: oxidize NADH to NAD^+^ and decrease the ratio of NADH/NAD^+^) improved it. Because these genes functions were results of homologous blast, they may perform opposed functions in ZM4. Unfortunately, the maximum WV decreased with addition of nicotinic acid or nicotinamide and overexpressing redox-related genes. Owing to *Z. mobilis* sensitive to the intracellular redox change, altered ratio of NADH/NAD^+^ possibly inhibited electricity performance. Therefore, maintaining redox homeostasis is the precondition for improving voltage output of ZM4. Besides, this fact emphasized that if gene manipulation was adopted to enhance electricity performance, keeping redox balance should be focused, because the voltage improvement resulted from gene manipulation may be counteracted by the negative effect of redox imbalance during this modification process.

### Improvement of MFC by the strategies combination

**Fig. 9:**
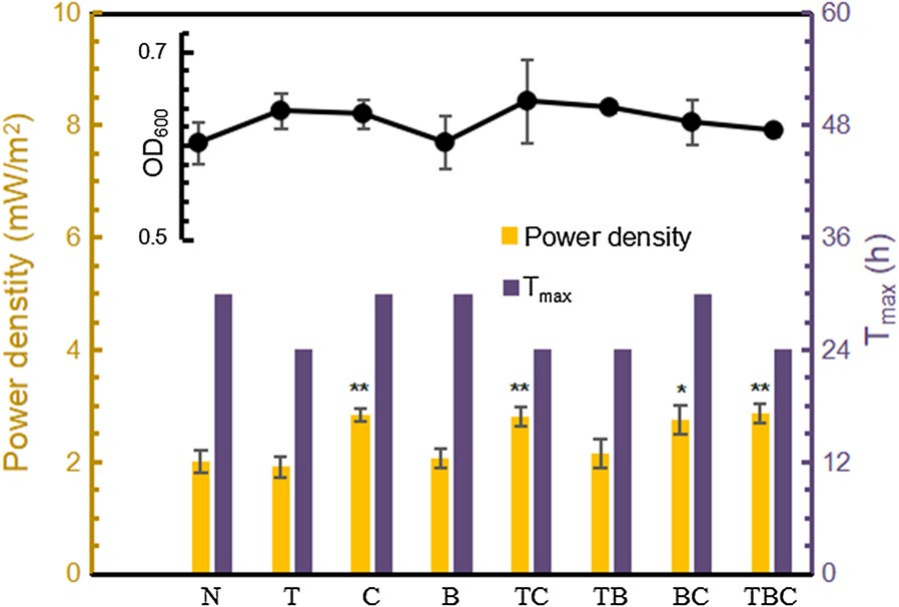
Maximum power densities and time needed to reach WV peaks (T_max_) in different treatment methods (N: no treatment, T: addition of Tween 80, C: addition of *c*-type cytochrome, B: biofilm removal). The combination of these letters means the cooperation of various methods and the black line on the top of the figure stands for the biomass of different treatment methods. *p* values were calculated between groups with and without treatment to compare the difference of electricity performance (*p value < 0.05; **p value < 0.01)

To further upgrade voltage output, cooperation of several effective methods (biofilm removal, addition of *c*-type cytochrome and Tween 80) were also tested. Figure 9 reveals that with addition of *c*-type cytochrome, electrical energy increased by 30% (from 2.0 to 2.6 mW/m^2^) and Tween 80 helped reduce the time needed to reach the WV climax for MFC by about 6 h. Any treatment involving these two methods obtained the positive effect on electricity generation. However, biofilm removal did not affect MFC obviously. As the previous biofilm removal results, only repeated batch fermentation was able to influence WV in *Z. mobilis*-inoculated MFC. Enough accumulation and long-term growth leads to more dead cells trapped in the biofilm, thus refreshing the biofilm with short fermentation time cannot exhibit significant improvement of electricity production. Therefore, combination of *c*-type cytochrome and Tween 80 improved power density and accelerated the entire electricity generation simultaneously showing that advantages of these two methods could be superposed to benefit electricity performance more.

**Fig. 10:**
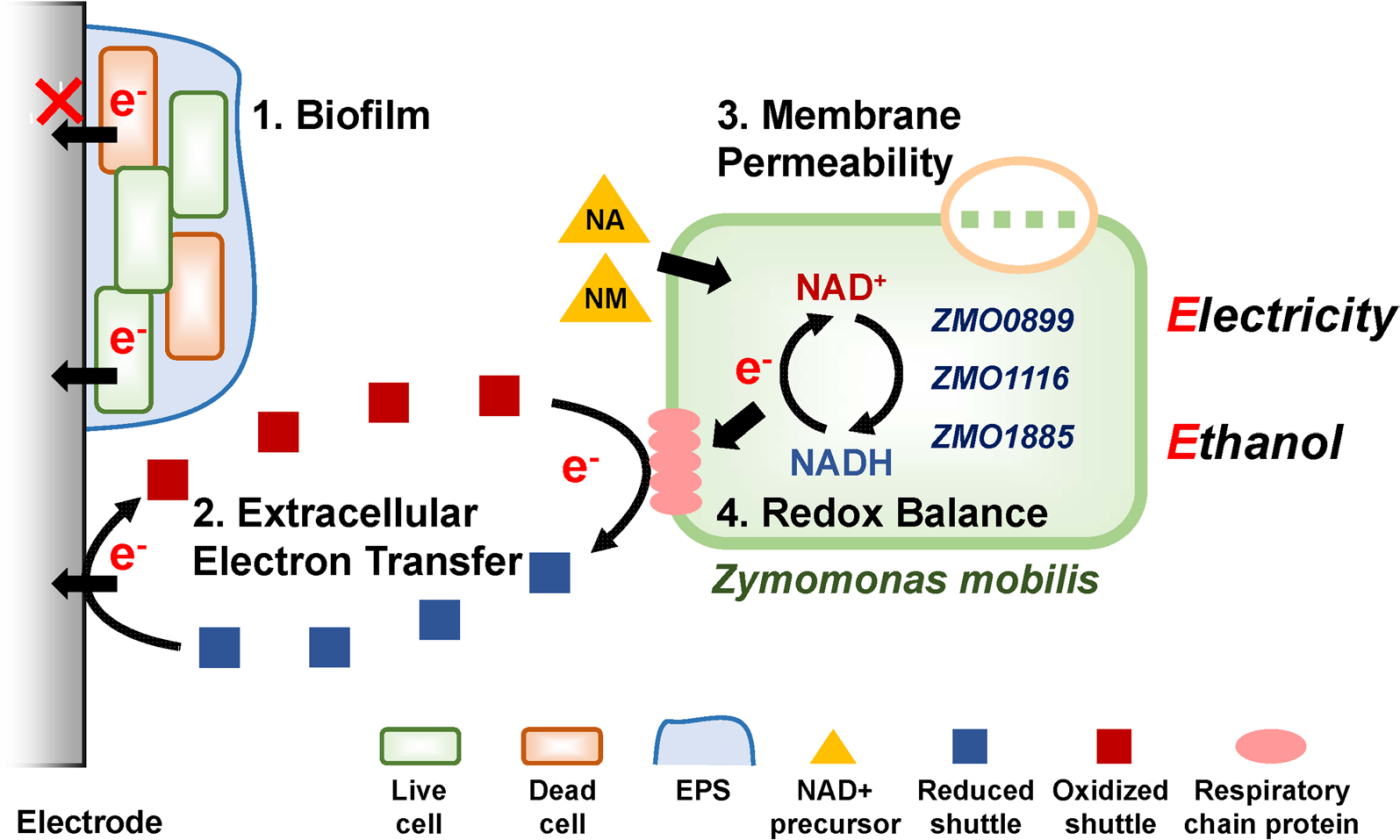
Schematic diagram of extracellular and intracellular strategies implemented in MFC

In summary, the strategies and related mechanisms are shown in Fig. 10 to demonstrate the outline of the designs in this paper.

## Conclusions

A *Z. mobilis*-inoculated bio-electrochemical system was constructed, which was directly driven by ORP difference and obtained a considerable bioelectricity generation (2.0 mW/m^2^). Two types of electricity generation were observed: glucose-independent process and glucose-dependent process. Several strategies were implemented to improve the performance. Biofilm removal and *c*-type cytochrome helped cells to promote electricity generation and Tween 80 accelerated voltage output. Redox balance was identified to be the precondition for higher electricity output. The combination of multiple methods realized further improvement of power. Overall, *Z. mobilis* was proven to be a promising strain for energy production in an efficient and environmentally sustainable manner.

## Methods

### Strains, medium, and cultivation

Wild type and genetic manipulated *Z. mobilis* ZM4 were cultivated statically at 30 °C for seeds. A loop of colonies was scratched from solid plate and inoculated into 100 mL RMG2 (g/L): 10 yeast extract, 2 KH_2_PO_4_, and 20 glucose. After 18 h, 14 mL OD_600_ = 1 culture was transferred into 126 mL RMG10 (g/L): 10 yeast extract, 2 KH_2_PO_4_, and 100 glucose. 20 μg/mL tetracycline was added in RM medium if using engineered strains to prevent the loss of expression vectors.

### MFC setup and ORP monitoring

Carbon cloth was used as the electrodes for both anode (2.5 cm × 2.5 cm) and cathode (2.5 cm × 3 cm). The H-type MFC was separated by Nafion 117 membrane, which was pretreated in 1 mol/L HCl for overnight and kept in sterile distilled water before H-type MFC setup. 140 mL K_3_[Fe(CN)_6_] (50 mmol/L) and RMG10 were supplemented to cathode chamber and anode chamber, respectively. The MFC was incubated at 30 °C statically. The open circuit voltage (OCV) and the work voltage (WV) with a 2 KΩ resistor were recorded. The data reported is the average from triplicates, with error bars representing standard deviations. An ORP electrode (model: Pt4805-DPASSC-K8S/225, Mettler Toledo, Switzerland) was inserted into the fermentor to monitor ORP of medium, and a handheld ORP monitor (MT-8050) was used to measure ORP in MFC.

### Quantification of metabolites

The sample was centrifuged at 8000 rpm for 2 min to collect the supernatant, which was used to quantify glucose and ethanol by HPLC (Waters 1525) with a column (Aminex HPX-87 H 300 × 7.8 mm, Bio-Rad, USA) and an RI detector (Waters 2414). The mobile phase consisted of 4 mmol/L H_2_SO_4_, and the flow rate was set at 0.6 mL/min. The temperatures for column and RI detector were set at 50 °C.

### Bio-electrochemical analysis

Cyclic voltammetry (CV) was performed on a CHI 1000C multichannel potentiostat (CH Instrument, Shanghai, China) and was conducted on a three-electrode configuration with the anode as the working electrode, the cathode as the counter electrode, and the Ag/AgCl (vs. SHE) as the reference electrode. Different resistors were used to obtain the maximum power density and the polarization curves. Electrochemical impedance spectroscopy (EIS) was performed to evaluate the internal resistance by using the same three-electrode system as CV experiment. Alternating current potential (maximum amplitude of 10 mV) in the frequency range of 100 kHz–5 MHz was applied to EIS experiments and the results were plotted in the form of Nyquist curve (Additional file 1: Figure S1) where the charge transfer resistance (R_ct_) was calculated by Z view software.

### Energy flow calculation

Combustion heat was calculated to represent the energy stored in substances. The combustion heat of glucose and ethanol are − 2804 kJ/mol and − 1366.8 kJ/mol, respectively. The combustion heat of biomass and yeast extract was estimated by Mendeleev’s empirical formula. Their elemental compositions were measured by Elemental Analysis (Flash Smart, ThermoFisher, China). After calculation, the estimated combustion heats of biomass and yeast extract were − 18,654.11 kJ/kg and − 15,473.58 kJ/kg, respectively. The electrical energy released by MFC was calculated by integrating the power density with time.

### Quantitative analysis of biofilm

Six wells plates were used to hold carbon cloth as platform for biofilm formation. After cultivated for 0, 6, 30, 60 h, carbon clothes with biofilm were picked out and soaked in crystal violet for 20 min, and then were rinsed with water for two times and dissolved in 1 mL 95% ethanol for OD_595_ measurement. Biofilm attaching electrodes was collected at 0, 6, 30, and 60 h to test their viability with LIVE/DEAD BacLight™ Bacterial Viability and Counting Kit. Viability was measured by counting the live cells (green dots) and dead cells (red dots) and calculating the ratio according to Additional file 1: Figure S2. NADH/NAD^+^ ratio at 24 h was obtained by Beyotime™ NAD^+^/NADH measurement kit.

### Addition of reagents

The mediators were added in the medium at the start of bacterial growth to test their interactions with *Z. mobilis* in MFC. Also, for electron shuttles, the control experiments with mediators were performed by adding these mediators in the medium without *Z. mobilis* to measure the electricity performance of these mediators by themselves.

Electron shuttles, surfactant, cytochrome protein and NAD^+^ precursors are shown in Table 1.

**Table 1:** The chemicals used in this study

Name	Sort	Concentration
Methylene blue	Electron shuttle	1, 5, 10 mmol/L
Neutral red	Electron shuttle	1, 5, 10 mmol/L
Humic acid	Electron shuttle	1, 10, 20 mg/L
1,4-Butanedisulfonate	Electron shuttle	1, 5, 10 mmol/L
Methyl naphthoquinone	Electron shuttle	1, 5, 10 mmol/L
TEMPOL	Electron shuttle	1, 5, 10 mmol/L
Riboflavin	Electron shuttle	1, 5, 10 mmol/L
*c*-type cytochrome	Cytochrome protein	0.1, 0.5, 1 mmol/L
Tween 80	Surfactant	5, 20, 80 mg/L
Nicotinic acid	NAD^+^ precursor	1, 10 mmol/L
Nicotinamide	NAD^+^ precursor	1, 10 mmol/L

### Mathematical analysis

Scaling values is a normalization to restrict range of values between 0 and 1, such as values of WV and glucose consumption rate:

1 $${\text{Scaled}}\;\;V = (V - V_{\text{min} } )/(V_{\text{max} } - V_{\text{min} } ).$$

The improvement ratio of OCV and WV based on electron shuttle or cells were adopted to estimate their effects on electricity generation.

2 $$R_{\text{or}} = \left( {O - O_{\text{S}} } \right)/O_{\text{C}} ,$$

3 $$R_{\text{wr}} = \left( {W - W_{\text{S}} } \right)/W_{\text{C}} ,$$

4 $$R_{\text{o}} = \left( {O_{\text{S}} - O_{\text{C}} } \right)/O_{\text{C}} ,$$

5 $$R_{\text{w}} = \left( {W_{\text{S}} - W_{\text{C}} } \right)/W_{\text{C}} ,$$

6 $$R_{\text{os}} = \left( {O - O_{\text{R}} } \right)/O_{\text{C}} - R_{\text{o}} ,$$

7 $$R_{\text{ws}} = \left( {W - W_{\text{R}} } \right)/W_{\text{C}} - R_{\text{w}} .$$

*R*_o_ and *R*_w_ are OCV and WV improvement ratio of cells. *R* with subscript combination (*R*_or/wr_ and *R*_os/ws_) is voltage improvement ratio of reagents and cells with reagents, respectively. The single characters (*O* and *W*) are the maximum voltage (OCV and WV) of cooperation of reagents and cells. The characters with subscripts—R, S, C are maximum voltage of reagents, cells and medium, respectively.

### DNA preparation, manipulation, and transformation

According to database in Kyoto Encyclopedia of Genes and Genomes (KEGG), the genes involved in cofactor (NADH/NAD^+^ and NADPH/NADP^+^) were selected to overexpress in *Z. mobilis* ZM4, including *ZMO0899* (NAD^+^ synthase), *ZMO1116* (glutamate synthase) and *ZMO1885* (NADH oxidase). The sequences are shown in Additional file 1: Table S2. These genes were amplified from ZM4 genome and ligated with native gap prompter and linearized vector pHW20a plasmid through homologous recombination. The newly constructed plasmids were identified and confirmed by PCR, restriction endonucleases digestions, and DNA sequencing. After DNA demethylation, the vectors were successfully transformed into electro-competent *Z. mobilis* cells by electroporation through an electrical pulse created by Gene Pulser^®^ II Electroporator (Bio-Rad) set at 25 μF, 1.60 kV, and 2000 Ω [30]. The result of PCR verification was demonstrated in Additional file 1: Figure S3.

## Supplementary information

**Additional file 1.** Additional figures and tables.

## Data Availability

All data generated or analyzed during this study are included in this published article and supplementary materials.

## Abbreviations

MET: Microbial electrochemical technologies
MFC: Microbial fuel cell
ORP: Oxidation–reduction potential
EET: Extracellular electron transfer
R_ct_: Charge transfer resistance
NA: Nicotinic acid
NM: Nicotinamide
NADH: Nicotinamide adenine dinucleotide
OCV: Open circuit voltage
WV: Work voltage
CV: Cyclic voltammetry
EIS: Electrochemical impedance spectroscopy
