# Supplementary material for: Potential of Zymomonas mobilis as an electricity producer in ethanol production
Source: Biotechnol Biofuels. 2020 Mar 5;13:36. doi: 10.1186/s13068-020-01672-5 (PMC7057670; doi:10.1186/s13068-020-01672-5)
Supplement: Supplementary file 1 — Additional file 1. Additional figures and tables. [file 13068_2020_1672_MOESM1_ESM.docx]

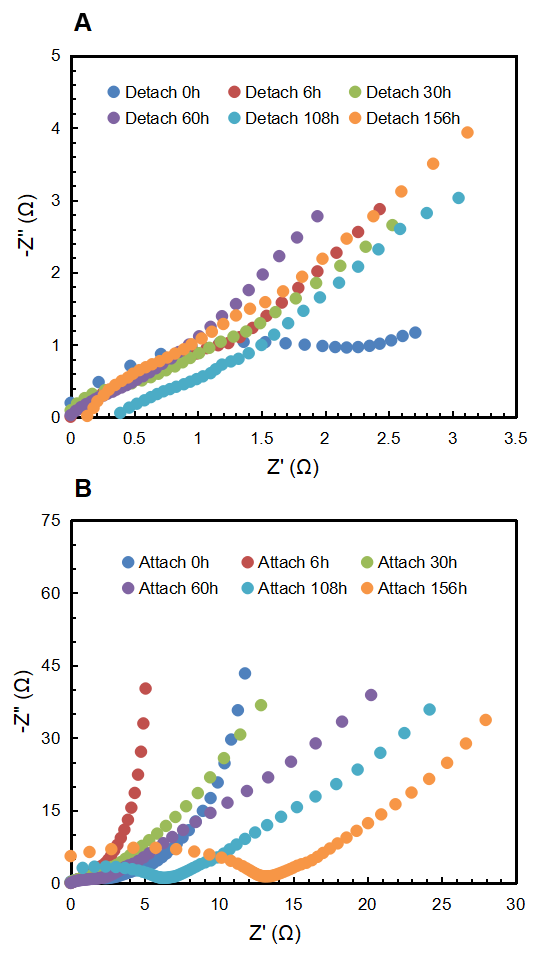


**Figure S1** Original EIS data of the electrodes in detachment group (**A**) and attachment group (**B**) at various time.


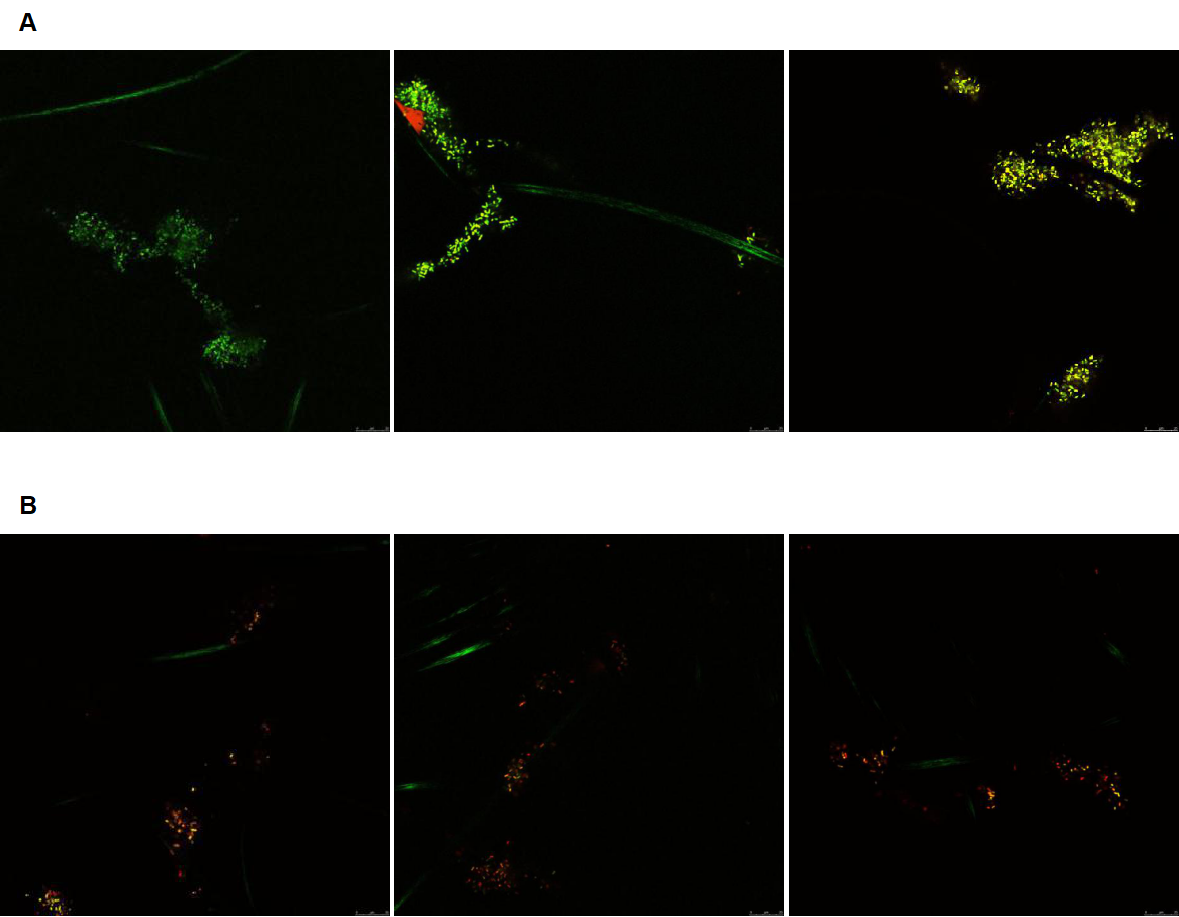


**Figure S2** Fluorescence microscope images of electrode surface

at 30 h (A) and 60 h(B). For each time point, three photos at different areas of biofilm were shown.

**
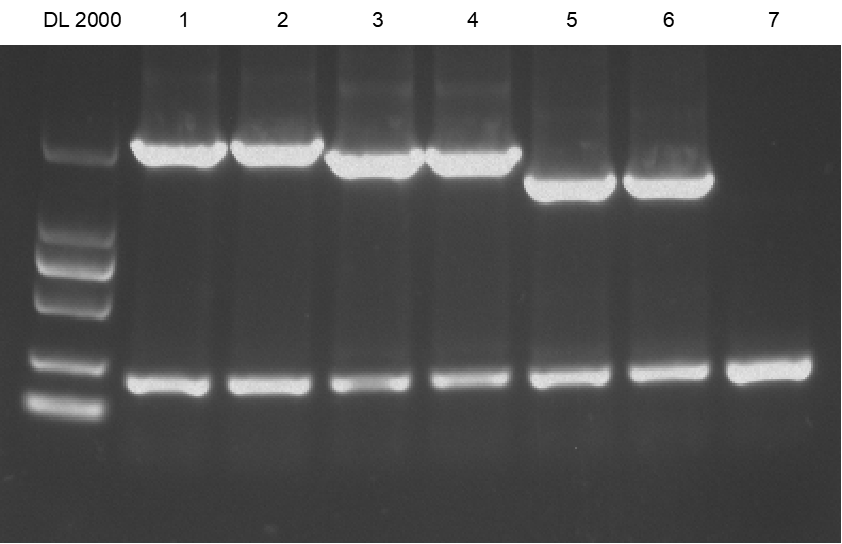
**

**Figure S3** Verification of PCR products on agarose gal (1: *ZMO0899*; 2: *ZMO0899* positive control; 3: *ZMO1116*; 4: *ZMO1116* positive control; 5: *ZMO1885*; 6: *ZMO1885* positive control; 7: Negative control)

**Table S1** O, O_R_, W and W_R_ of reagents at three different concentrations(1:lower concentration; 2:middle concentration; 3:higher concentration).

| Reagent | Symbol | 3 | 2 | 1 |
| --- | --- | --- | --- | --- |
| Methyl naphthoquinone | O (mV) | 638.1±13.1 | 609.7±8.4 | 590.2±5.8 |
|  | O_R_ (mV) | 615.7±10.2 | 582.4±11.3 | 572.9±2.8 |
|  | W (mV) | 80.4±1.2 | 73.6±3.4 | 61.6±2.2 |
|  | W_R_ (mV) | 75.3±2.4 | 70.3±1.7 | 57.7±2.1 |
| 1,4-butanedisulfonate | O (mV) | 608.3±2.9 | 555.7±3.7 | 521.1±10.1 |
|  | O_R_ (mV) | 589.8±2.8 | 561.3±7.9 | 569.7±1.8 |
|  | W (mV) | 75.8±1.7 | 71.4±2.7 | 63.7±2.9 |
|  | W_R_ (mV) | 70.8±0.7 | 66.7±1.4 | 58.6±0.4 |
| Methylene blue | O (mV) | 581.1±7.8 | 572.1±8.9 | 530.9±7.9 |
|  | O_R_ (mV) | 561.8±8.9 | 553.2±7.6 | 540.7±5.8 |
|  | W (mV) | 237.2±4.8 | 184.8±5.8 | 120.5±6.1 |
|  | W_R_ (mV) | 212.7±8.7 | 160.4±1.8 | 99.8±2.3 |
| Neutral red | O (mV) | 838.1±10.8 | 784.2±4.2 | 712.2±5.7 |
|  | O_R_ (mV) | 825.1±4.8 | 768.4±7.5 | 692.9±3.7 |
|  | W (mV) | 78.5±0.8 | 73.4±3.1 | 68.9±3.5 |
|  | W_R_ (mV) | 71.9±1.2 | 68.2±0.8 | 62.4±6.1 |
| Riboflavin | O (mV) | 553.1±2.8 | 546.8±1.2 | 537.3±3.4 |
|  | O_R_ (mV) | 531.3±4.1 | 548.7±8.6 | 539.6±1.1 |
|  | W (mV) | 61.2±0.6 | 63.4±3.1 | 62.7±1.9 |
|  | W_R_ (mV) | 54.2±1.2 | 60.3±1.7 | 58.5±1.2 |
| TEMPOL | O (mV) | 271.3±2.4 | 284.7±6.8 | 282.9±3.9 |
|  | O_R_ (mV) | 259.6±5.8 | 274.5±7.8 | 279.6±3.2 |
|  | W (mV) | 39.8±1.7 | 40.2±2.4 | 41.9±0.7 |
|  | W_R_ (mV) | 31.5±2.4 | 32.4±0.5 | 31.7±0.6 |
| Humic acid | O (mV) | 501.7±2.1 | 486.2±8.4 | 492.4±5.1 |
|  | O_R_ (mV) | 472.3±4.5 | 455.3±2.8 | 461.2±3.3 |
|  | W (mV) | 79.2±1.1 | 75.1±3.7 | 73.3±0.3 |
|  | W_R_ (mV) | 74.3±0.5 | 71.3±0.7 | 69.8±1.1 |
| *c*-type cytochrome | O (mV) | 518.1±13.1 | 512.7±8.4 | 509.2±5.8 |
|  | O_R_ (mV) | 275.7±10.2 | 272.4±11.3 | 274.9±2.8 |
|  | W (mV) | 59.4±0.3 | 57.3±3.9 | 58.1±2.1 |
|  | W_R_ (mV) | 25.6±2.1 | 26.1±1.2 | 23.1±0.9 |

**Table S2 Gene name and related sequence**

| Gene name | Gene sequence |
| --- | --- |
| *ZMO0899* | atgagcatga ctgaccaatt gacaatactg ttagctcaaa tcccgcaaac ggtcggtcat  tgccgccgta atgcggatgc tatgctgaaa atcaggcaag aagcggggaa ggttgatctt  atcctgtttc ctgaattgca attgatcggt tatcctcccg aagatctcgt tttaaagccg  tctttattgc aaaatgccaa ggaagaattg gagcgtttgg ctcttgcgac atctgatggc  ggggcggcaa tgttggttgg aactgcgtgg caggaaaagg ggcggatttt caatgccgtt  gccttgctgg atcaaggtaa aattgcggct attcgctata aacatgactt gccaaattac  ggcacctttg acgaaaaacg gattttttca gcggggcctc ttcctgaacc tgttttgttt  agagatgtct ctcttggtat tcctgtttgt gaagatatct ggacagaaaa actctgtgct  catctgaaag aacgcggagc ggaaatactt cttgtgccga atggcagtcc ttttgaaagt  ggtaaatatc cggttcggca taaagttgtt gcagatcggg ttaaagaaac aggattgccc  tgcctgtatc tgaacagggt cggtggacag gatgaagtcg tttttgatgg tggctctttt  gttgtcaatc gagatggtgc agtgcctatc tctctgccgc aatggcaggc ggattgttat  gtaacccatt ggcataagac ggaagaaggt tggtcttgtg atgagggttt acagagtaaa  atctcagaag gtgaagaccg gcgattaagc gatatttatc aagccatgat gatggggctt  ggcgattatg tccgccagaa tggtttttct ggtgttttgc tgggcttatc cggtggtatt  gacagcgcgc tttcggcggc ggtcgcagta gatgcccttg gtgcagataa agtctggtgc  gtgatgctgc cttcccgcta taccagtcaa gaaagcctcg atgatgcgcg cgcttgtgct  gagatgctgg gatgtcggtt agattctgtt cccatcaatc cggcagttga agctattgac  gggatgctgg ctccactttt ttcagggaaa aagcctgata ttaccgaaga aaacattcaa  tcccgtttgc gcggggtcgt gttaatggcg atttccaata aattttctcc aatggtgtta  accacgggaa ataaaagtga aatggccgtt ggctatgcga ccctttatgg cgatatgaat  ggcggctatt ctgtcctgaa agatctttat aaaagtgatg tctttaaggt gtcccgttgg  agaaatagcc ataaaccggc ccatgcttta ggccccgatg gctatgtcat gcccgatcga  gtgattacca agccaccttc cgcagaattg cgggaagatc aaaaagatag cgattcatta  cctccttatg atatattgga tgctgtcttg catggcttga ttgaagagga tttatcctgt  tctgatctgg tcaaacgcgg ctatgatcct gatttggtca cgcggattga aaatctgctc  catcgtgccg aatataaaag aaggcaggct ccccccggtg tgaaactggg tagccgcaat  ttcgggcggg atcgccgtta tccgctttcc catcaatttc gttcttctca ttcctga |
| *ZMO1116* | atggcgcaaa ataaaatgct gtcctttgtt catctgaaac aggcctatcc tgaaaaacga  tcagcacagg aaagagacaa agactttaac gagatttatc ggtctttctc tgacgaacag  gcgagctgtc aggcatcccg ttgttctcaa tgtggggtgc cttattgttc agcttactgt  ccgctgcata acaatattcc agactggcta aaaatgacgg cagaaggccg gttgcaggat  gcttatgccc tttccagtgc aacctcgacc atgccggaaa tttgtggccg catttgcccg  caagaccgtc tttgtgaagg taactgcgtt attgagaatt ccggtcatgg cgctgtaacc  attggatcgg ttgaaaaata catcaccgat acggcatgga aagaaggttg ggttgaaccc  ttacagccta taactgaact tggtatttcg gttggtatta tcggttcagg gcctgccgga  ttgacggcgg ctgaaaaatt acggcagcaa gggtatcagg tgcatgttta tgatcgccat  gatcgtgccg gtggcctttt gacctatggt attccgggct tcaaactcga aaaagacatt  gttgcacgcc gtgttaaacg gttagccgat gggggtgtca ccttccatac caatttccac  gtcggtaaag atgccagcct tgaagaattg cgtgagcgcc acgcggctat tctgatcgca  accggtgttt ataaagcccg cggtctggct acccccggaa aagaattaaa cggtattgtt  gatgcgctgg atttcctgat tagctccaac cggaaatctt ttggtgacaa ggtcgaagcc  tttgataatg gcacccttga tgccaagggt aaaaaagtag tggttatcgg tggtggtgat  accgcaatgg actgcgtcag aacggctatc cgtcagggtg cagaatctgt ccgctgcctc  tatcgtcgcg atcgagtcaa tatgcccggt tccgcacgcg aagtaaaaaa tgccgaggat  gaaggcgcag aatttgtctg gctatctgca ccggaatctt ttgtcggaaa agaccatgtt  caatctgtaa aagtccgtgg aatgcgcctg ggtgcgccag atggttctgg ccgtcgttct  cctgaacctg atccagcacg tctttttgac atggatgcgg atcttgtcat ttgcgctttg  ggtttttcac cggaagacct gccaacgctg ttcaatgcgc cggaattatc ggttacccct  tggggaaccg tagaggctga tccggtcaca ttacagacta gcatcgaagg ggtctttgct  gctggtgata ttgtccgcgg tgccagcctt gtggtttggg ccatccgtga cggtctggtc  gcaagcgaac agatgcatca atggctagca acccgccagc aggacaaaca ggacgcaccg  acaaaggcta aaaagaaagc tgtttttgcc tga |
| *ZMO1885* | aatcacatct ttggtaaggg gggctttctc aaagaaacgc cgtggcaaat caggattacc  gataaaaggc cttccaaaac tgatggcatc ggccacaccg gaatcaagag cagcttgcgc  ggtttcaaaa gtataatcct gattaagaac caaaggcggt ttgaaaactt ttcggatttc  aggcgataat ttgggctgat ctgttttgcc aaaggtgcca tcaacagccc cttctcgcat  ccctaaaaag gcaatatcca aatcagacaa cattttggcc gccggtataa aaacctgttc  gggatgacta tcaaccgtcc cctgtatttc accattcggt gataacctaa cggccgtccg  ctctttccca atggttgcaa taactcgttc ggtgacgtct ttcagcaaac gaatacggtt  ttcaactgca cccccatatt catcatgacg atggttggtg ctatcccgta taaattcgtc  aatcaaataa ccattagcgg catggatctg tacgccatca aaaccggcct tcaaggcatg  acgggcagcc ttttcataat catcaagaag acgcggaatt tcatccaatc gcaaagcgcg  ggcaacatca taaggctttt taccatcata ggtatgcccc aatccgggtg cctgactggc  agaaggcgca acaggctgca tcccactgac attagacggc accatacgtc ccatatgcca  tagctgggca aagataagac cgccggcatc atgcacggcc tgcgtaatcg gcagccaggc  ttctacttgg gcatcactcc agattcccgg agcataaggc cagcccaaac cttcctgact  aatgccagtc gcttctgaaa taatcagtcc ggcgcttgcc ctctgggcat aatattcggc  cattatttca gtggggacat gatcgcgcgt ggcacggcca cgggtcagag gcgccatcca  aattctattt tttgcggtaa aagcgccgaa gcggatggga tcaaacaagc taggcat |
